# Chromosomal radiosensitivity of human breast carcinoma cells and blood lymphocytes following photon and proton exposures

**DOI:** 10.1007/s00411-023-01016-5

**Published:** 2023-02-10

**Authors:** Agata Kowalska, Elena Nasonova, Polina Kutsalo, Konrad Czerski

**Affiliations:** 1 Institute of Mathematics, Physics and Chemistry, Maritime University of Szczecin, Wały Chrobrego 1, 2, 70-500 Szczecin, Poland; 2 Joint Institute for Nuclear Research, Joliot-Curie 6, 141980 Dubna, Russia; 3 Institute of Physics, University of Szczecin, ul. Wielkopolska 15, 70-451 Szczecin, Poland

**Keywords:** Breast cancer cells, Proton radiotherapy, Chromosome aberrations, Linear-quadratic model, Premature chromosome condensation

## Abstract

Breast carcinomas (BC) are among the most frequent cancers in women. Studies on radiosensitivity and ionizing radiation response of BC cells are scarce and mainly focused on intrinsic molecular mechanisms but do not include clinically relevant features as chromosomal rearrangements important for radiotherapy. The main purpose of this study was to compare the ionizing radiation response and efficiency of repair mechanisms of human breast carcinoma cells (Cal 51) and peripheral blood lymphocytes (PBL) for different doses and radiation qualities (^60^Co γ-rays, 150 MeV and spread-out Bragg peak (SOBP) proton beams). The radiation response functions obtained using the conventional metaphase assay and premature chromosome condensation (PCC) technique enabled us to determine the number of chromosomal breaks at different time after irradiation. Both cytogenetic assays used confirmed the higher biological radiosensitivity for proton beams in tumor cells compared to PBL, corresponding to higher values of the linear LQ parameter α. additionally, the ratio of the LQ parameters β/α describing efficiency of the repair mechanisms, obtained for chromosome aberrations, showed higher numbers for PBL than for Cal 51 for all exposures. Similar results were observed for the ratio of PCC breaks determined directly after irradiation to that obtained 12 h later. This parameter (t0/t12) showed faster decrease of the repair efficiency with increasing LET value for Cal 51 cells. This finding supports the use of the proton therapy for breast cancer patients.

## Introduction

DNA double-strand breaks (DSBs) are the most hazardous DNA lesions induced by ionizing radiation, causing the loss of genetic integrity and formation of chromosome aberrations (CA). Knowledge of the induction of DSBs in healthy and cancer cells and the efficiency of repair mechanisms is crucial in understanding of the biological response to different radiation qualities and success of the cancer radiotherapeutic treatment plans.

In our previous studies, we have applied the conventional metaphase assay to characterize the chromosome aberration (CA) yield induced in human peripheral blood lymphocytes (PBL) by protons and heavy ions (Kowalska et al. 2017, 2019; Czerski et al. 2019). This method allows the detection of the final residual damage in dividing cells. In order to study the induction and the repair efficiency of DSBs leading to CA, we have also applied the premature chromosome condensation (PCC) technique, which enables to condense chromosomes in interphase and visualize genetic damage at selected moments of time, i.e., shortly after the exposure or after repair completion (Kowalska et al. 2020).

In the present work, we would like to complete our previous CA and PCC studies performed in PBL exposed to protons and gamma rays and compare them to new results obtained for human breast carcinoma cell line Cal 51 (SIB 2021). Up to now, multiple studies were reported on CA in PBL (George et al. 2015; Schmid et al. 1997; Cornforth et al. 2017) while the conduction of the research on Cal 51 seems to be very valuable. This highly tumorigenic cell line is characterized by a very stable diploid karyotype without numerical aberrations and very low level of spontaneous CA (Gioanni et al. 1990; Davidson et al. 2000). In contrast, the vast majority of breast cancer (BC) cell lines established to date, as well as other tumor cell lines, are highly abnormal, carrying rearranged chromosomes and numerical changes (Davidson et al. 2000). Thus, Cal 51 provides a valuable model of tumor cells in comparative cytogenetic studies of radiation action.

Breast carcinomas are among the most frequent cancers in women. They are very heterogeneous and can be divided into distinct subtypes (Kao et al. 2009; Chavez et al. 2010) with different radiosensitivity. Cal 51 tumor cells, used in this study, belong to Basal B subtype, triple-negative BC (TNBC) without hormone receptors and HER-2/Neu amplification (Chavez et al. 2010) and are associated with poor prognosis. Studies on radiosensitivity and features of radiation response of TNBC are scarce (Masoudi-Khoram et al. 2020) and are mainly focused on intrinsic and/or acquired radioresistance and molecular mechanisms underlying it, in order to develop effective treatment modalities (Zhou et al. 2017; Gray et al. 2019, 2020). These investigations provided only few data for successful comparison of tumor and normal tissue cell radiosensitivity, which is also of clinical importance (Peters 1990). To our knowledge, there are no data on chromosomal radiosensitivity of TNBC cells, or on their response to different radiation types, particularly γ rays and protons, which currently are widely used in their treatment. The detailed knowledge about differences in effectivity of repair mechanisms and the resulting chromosomal radiosensitivity of tumor and surrounding normal cells may significantly improve clinical outcome of radiotherapy.

For the practical purposes, CA formation as a radiation response is usually presented in the frame of the linear-quadratic (LQ) model:

1 $$Y = Y_{0} + \alpha \cdot D + \beta \cdot D^{2}$$

where ${Y}_{0}$ is the constant term for the background and *α* denotes the linear parameter describing the damage probability of the irradiated cells that directly depends on the LET value and the local ionization density of the utilized radiation (Ando and Goodhead 2016). In contrast, the quadratic term *β* reflects the efficiency of the biological repair mechanisms, especially at lower doses, for which the physical effect of overlapping ion tracks is relatively weak (Kowalska et al. 2017). The same model can be also applied for a direct study of the chromosome breakage using the PCC technique. Thus, in contrast to CA, which can be observed only after completion of a full cell cycle, the PCC method allows to visualize genetic damage at any selected time after the exposure. Consequently, efficiency of the repair mechanisms can be studied not only by means of absolute parameter values of the LQ model, but also using their temporal change. This is particularly important in view of different dynamics of the two main repair mechanisms: *nonhomologous end joining* (NHEJ) and *homologous recombination* (HR). While the first one, lasting up to several minutes, is predominant in the fast, initial component of the DSBs repair, the second one is more time-demanding and takes many hours (Khanna and Jackson 2001; Budman and Chu 2005; Grosse et al. 2014). As already presented in our previous investigations (Kowalska et al. 2020), the appropriate selection of the measurement time points at a large time distance can help us to focus on the second repair component mechanism and to compare it with CA results.

In this study, the experimentally determined distributions of CA and PCC breaks as well as the fitted parameters of the LQ model obtained for both PBL and Cal 51 cells after exposure to the therapeutic proton beams and γ-rays will be exploited to discuss advantages and limitations of the applied methods for investigation of cellular repair mechanisms. The LQ model will be also used to determine relative biological effectiveness (RBE) of protons in human lymphocytes and Cal 51, which is especially interesting for proton therapy, for which a constant value of 1.1 is usually assumed. Due to the applied PCC technique, data obtained at two different time points after irradiation can be presented.

## Materials and methods

### Cell lines and culture

The whole blood used for the study was obtained by venipuncture into heparinized vacuum containers. The samples were collected from informed, healthy volunteers, in accordance with local ethical regulations. For the conventional metaphase assay, the whole blood samples (including resting lymphocytes at G_0_ cell cycle stage) were irradiated in 0.5 ml Eppendorf tubes. For PCC analysis, the lymphocytes were isolated by gradient centrifugation and seeded with a density of 0.5 × 10^6^ cell/ml in the in RPMI medium supplemented by 20% fetal calf serum, 2 mM L-glutamine, 100 U/ml penicillin, 100 μg/ml streptomycin and 1% phytohaemagglutinin (PHA). After 48–60 h of culture, the asynchronously growing lymphocyte population was exposed in suspension to ^60^Co γ-rays and protons (150 MeV and SOBP) in the culture flasks.

Human breast carcinoma cells Cal 51 were maintained in Dulbecco’s modified minimal essential medium (DMEM) supplemented with 10% fetal calf serum, 2 mM L-glutamine and 1% penicillin/streptomycin (all reagents from Sigma). Cells were stored at 37 °C in a 5% CO2 atmosphere. Asynchronously growing Cal 51 cells were irradiated as a monolayer in the culture flasks for both metaphase and PCC analysis. All exposures were done at room temperature, and controls were sham-irradiated.

### Irradiation

Proton exposure was performed at the clinical proton beam facility of the medico-technical complex of Dzhelepov Laboratory of Nuclear Problems, JINR, Dubna, Russia (for more details see Racjan et al. 2007 and Kubancak et al. 2013). Blood samples or Cal 51 monolayers in culture flasks were irradiated by an unmodified 150 MeV proton beam (LET 0.57 keV/µm) and by slowed down protons at the central region of the 10 mm wide plateau of the spread-out Bragg peak (SOBP) whose average LET of 1.4 keV/µm was determined experimentally (Kubancak et al. 2013). Dose rate in the target volume amounted to 0.7 Gy/min for high-energy protons and 1.3 Gy/min in the case of SOBP. As a reference, the ^60^Co γ-radiation source of the radiation therapy unit ROKUS-M was used. Dose rate at irradiation point was 0.82 Gy/min. Doses ranged between 0.5 and 5 Gy for metaphase assay and 0.5–2 Gy for PCC.

### Metaphase and PCC analysis

For metaphase analysis, after irradiation Cal 51 cells were allowed to grow in a complete medium at 37 ℃ and 5% CO_2_. Cells were fixed at 16 h after exposure, proceeded by a 1 h colcemid treatment (50 ng/ml) for metaphase accumulation, and stained with 3% Giemsa.

After exposure to proton beams and ^60^Co γ-rays the blood samples (resting PBL at G_0_ cell cycle stage) were diluted in 4.5 ml of RPMI medium supplemented by 20% fetal calf serum, 2 mM L-glutamine, 100 U/ml penicillin, 100 μg/ml streptomycin and 1.5% phytohaemagglutinin (PHA), incubated at 37° C and 5% CO_2_, fixed at 48 h after PHA stimulation, proceeded by a 3 h colcemid treatment (200 ng/ml) for metaphase accumulation, and stained with 3% Giemsa. Typically, 100–300 metaphases were analyzed for every data point. Chromosomal aberrations were classified according to Savage (1976). All aberrations of the chromosome and chromatid types visible without karyotyping were recorded. The chromosome-type aberrations comprise paired fragments, dicentrics, centric and acentric rings (the latter also includes double minutes) and translocations visible without karyotyping. The chromatid-type aberrations include the chromatid-type breaks and chromatid-type exchanges. The gaps were not scored as aberrations.

For the PCC analysis, PBL were isolated from the blood by gradient centrifugation using BD Vacutainer^®^ CPT^™^ (Becton, Dickinson and Co., USA) and cultured in the same RPMI medium 48 h prior to irradiation. Exponentially growing lymphocytes and Cal 51 cells were allowed to repair for various times after irradiation (0–12 h) and then were forced to condense chromatin prematurely by addition of 50 nM calyculin A (Sigma) immediately after irradiation and left for 1 h in 37° C. Then, the cells were treated with 0.075 M KCl for 10–15 min at 37 ℃ and fixed with methanol:glacial acetic acid (3:1). Cells were dropped onto a clean wet slide, airdried and stained with 3% Giemsa. Typically, 100–200 G_2_-phase cells were analyzed for every data point. The scoring and recording criteria followed those given in IAEA Manual (2011). The damage was classified as chromatid breaks, isochromatid breaks (excess figures) and chromatid exchanges (Kowalska et al. 2020). The yield of isochromatid breaks was measured from the excess number of chromosomes (> 46 figures) observed (IAEA 2011). In G_2_-phase of the cell cycle, the isochromatid break occurs when two breaks are formed on the opposite sister chromatids in a close proximity. Since one isochromatid break results from the breakage of both chromatid threads, one isochromatid break was scored as two chromatid breaks. Exchanges were also scored as two breaks. For further details, see (Kowalska et al. 2020).

### Statistical analysis of aberrations and chromosome breaks

Statistical distribution of the number of observed CA (or PCC breaks per cell) in human lymphocytes, as well as in Cal 51 cell line, was described by Poisson distribution. This stochastic distribution is used in the case of low-LET radiations for which the mean number of aberrations induced by a single particle transversal is low (Gudowska-Nowak et al. 2007). Thus, the energy imparted by many low-LET particles is almost homogenously distributed. For the simple Poisson statistics, the aberration frequency can be calculated as follows:

2 $$P_{p} \left( m \right) = \frac{{\lambda_{P}^{ } e^{{ - \lambda_{P} }} }}{m!}$$

Here, *m* stands for the number of aberrations per individual cell and* λ*_*P*_ is the average number of CA or chromosome breaks observed in the whole cell population exposed to a given dose of a given radiation.

## Results

### Metaphase assay

We found using mFISH that chromosome number (46, XX) and structure of Cal 51 cell line were markedly stable (data not shown). The level of spontaneous aberrations was 0.5–1%.

**Fig. 1:**
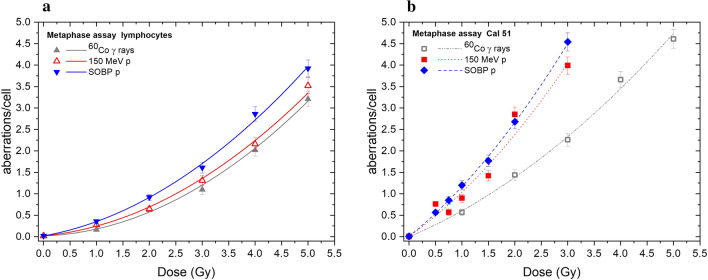
Dose dependence of CAs per cell induced by ^60^Co γ-rays, 150 MeV protons and SOBP protons (**a**) in human lymphocytes (data from Kowalska et al. 2019), (**b**) and in Cal 51 human carcinoma cells. Mean number of total aberrations per cell is shown. Error bars correspond to the statistical (Poisson) uncertainties

The percentage of aberrant cells, total CA yield and CA spectra in Cal 51 cells produced by photons and proton beams are listed in Table 1. Figure 1 compares the dose dependencies of CAs recorded in lymphocytes and Cal 51. For the lymphocytes, we used previously published PBL data (Kowalska et al. 2019). The aberration yields induced by all radiation species were higher for Cal 51 respect to PBL. The total aberration yields were fitted by a linear-quadratic function. Parameters of the fits are presented in Table 2. Uncertainties of *α* and *β* coefficients are given from the least squares regression. The uncertainties of *β/α* ratios were calculated using the total differential method.

**Table 1:** Frequency of CA induced in Cal 51 by ^60^Co γ rays, 150 MeV protons and SOBP protons

Irradiation	Dose, Gy	No. of cells scored	Aberrant cells (%)	Aberrations per 100 cells						Sum of aberrations/100 cells	Aberrations/aberrant cells
				ctb	csb	R ac	Dic	Rings	Trans/cte		
	*0*	*200*	*0*	*0.5*	*0.5*	*0*	*0*	*0*	*0*	*1*	*1.0*
	*1*	*100*	*33*	*6*	*23*	*4*	*16*	*2*	*6*	*57*	*1.7*
^60^Co	*2*	*100*	*67*	*15*	*55*	*8*	*18*	*6*	*42*	*144*	*2.1*
$\upgamma$ rays	*3*	*100*	*81*	*39*	*67*	*10*	*53*	*6*	*51*	*226*	*2.8*
	*4*	*100*	*92*	*54*	*96*	*16*	*62*	*12*	*126*	*366*	*4.0*
	*5*	*100*	*100*	*76*	*127*	*17*	*68*	*9*	*166*	*463*	*4.6*
	*0*	*200*	*0*	*0*	*1*	*0*	*0*	*0*	*0*	*0.5*	*1.0*
	*0.5*	*200*	*44*	*30.5*	*26*	*3.5*	*6*	*0.5*	*10*	*76.5*	*1.7*
150 MeV	*0.75*	*100*	*35*	*10*	*18*	*6*	*10*	*1*	*12*	*57*	*1.6*
protons	*1*	*100*	*50*	*22*	*21*	*10*	*17*	*2*	*18*	*90*	*1.8*
	*1.5*	*100*	*64*	*21*	*44*	*5*	*19*	*4*	*49*	*142*	*2.2*
	*2*	*100*	*94*	*82*	*87*	*12*	*21*	*3*	*80*	*285*	*3.0*
	*3*	*100*	*94*	*49*	*102*	*28*	*57*	*4*	*159*	*399*	*4.2*
	*0*	*200*	*0*	*0*	*1*	*0*	*0*	*0*	*0*	*0.5*	*1.0*
	*0.5*	*200*	*36.5*	*10*	*20*	*2.5*	*5.5*	*0*	*18.5*	*56.5*	*1.5*
SOBP	*0.75*	*100*	*48*	*15*	*30*	*4*	*11*	*2*	*23*	*85*	*1.8*
protons	*1*	*100*	*65*	*19*	*44*	*13*	*19*	*4*	*21*	*120*	*1.8*
	*1.5*	*100*	*76*	*32*	*52*	*20*	*23*	*5*	*45*	*177*	*2.3*
	*2*	*100*	*88*	*53*	*70*	*22*	*28*	*6*	*89*	*268*	*3.0*
	*3*	*100*	*95*	*73*	*92*	*23*	*44*	*2*	*220*	*454*	*4.8*

*ctb* chromatid breaks, *csb* paired fragments, *dic* dicentrics, *Race* acentric rings, *Rc* centric rings, *trans* translocations, *cte* chromatid exchanges

**Table 2:** Fitting parameters of CA dose–response curves for PBL (Czerski et al. 2020) and Cal 51

Irradiation	Lymphocytes			Cal 51		
	*α*	*β*	*β/α*	*α*	*β*	*β/α*
^60^Co γ rays	0.05 ± 0.03	0.12 ± 0.01	2.6 ± 1.8	0.52 ± 0.07	0.085 ± 0.018	0.16 ± 0.04
150 MeV protons	0.12 ± 0.03	0.11 ± 0.01	0.9 ± 0.3	0.87 ± 0.08	0.16 ± 0.04	0.18 ± 0.05
SOBP protons	0.22 ± 0.04	0.11 ± 0.01	0.52 ± 0.12	0.99 ± 0.08	0.17 ± 0.04	0.17 ± 0.05

The spectra of CA were also different: in Cal 51, 50% of the total aberration yield was accounted for by chromatid-type aberrations and about 50% were exchange-type aberrations, both of chromosome- and chromatid type. Irradiation of G_0_ PBL resulted in > 98% chromosome-type aberrations with 60–80% of exchange-type aberrations (Kowalska et al. 2019).

### PCC

**Fig. 2:**
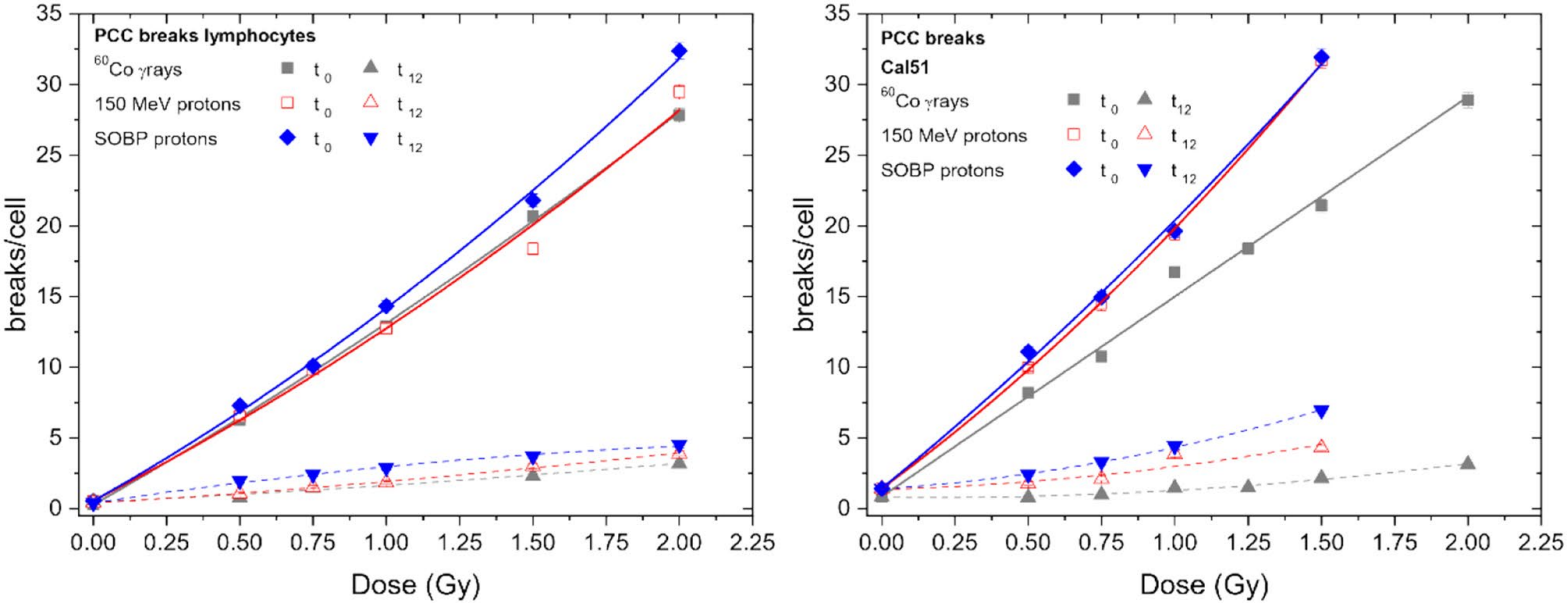
Dose dependence of PCC breaks per cell induced in human lymphocytes (left) (Kowalska et al 2020) and human carcinoma cells Cal 51 (right) by ^60^Co γ-rays, 150 MeV protons and SOBP protons at t0 (solid lines) and t12 (dotted lines)

Next, we evaluated the frequency of PCC breaks in Cal 51 and PBL as a function of dose of photon and proton exposures immediately (t0) or after repair completeness (t12) (Fig. 2) (PBL data taken from Kowalska et al. 2020) Parameters of the fits are presented in Tables 3 and 4.

**Table 3:** Fitting parameters of dose–response curves of PCC breaks measured for PBL (Kowalska et al. 2020) and Cal 51 at t0

Irradiation	Lymphocytes			Cal 51		
	*α*	*β*	*β/α*	*α*	*β*	*β/α*
^60^Co γ rays	11.8 ± 0.5	1.0 ± 0.3	0.09 ± 0.03	14.1 ± 0.2	–	–
150 MeV protons	10.6 ± 0.5	1.6 ± 0.3	0.15 ± 0.03	15.2 ± 0.6	3.2 ± 0.5	0.21 ± 0.03
SOBP protons	11.7 ± 0.5	2.0 ± 0.3	0.17 ± 0.03	16.9 ± 1.6	2.1 ± 1.4	0.12 ± 0.08

**Table 4:** Fitting parameters of dose–response curves of PCC breaks measured for PBL (Kowalska et al. 2020) and Cal 51 at t12

Irradiation	Lymphocytes			Cal 51		
	*α*	*β*	*β/α*	*α*	*β*	*β/α*
^60^Co γ rays	1.0 ± 0.2	0.19 ± 0.12	0.19 ± 0.13	0.3 ± 0.5	0.5 ± 0.2	2.1 ± 4.5
150 MeV protons	1.3 ± 0.2	0.26 ± 0.12	0.2 ± 0.1	0.6 ± 0.4	1.0 ± 0.3	1.7 ± 1.2
SOBP protons	3.1 ± 0.2	0.55 ± 0.14	0.18 ± 0.05	1.3 ± 0.4	1.6 ± 0.3	1.2 ± 0.5

The spontaneous level of PCC breaks was slightly higher in Cal 51 and accounted to 0.8–1.4 breaks/cell (breaks in untreated cells are considered to be an artifact of Calyculin A action). The initial breakage (t0) was similar in both cell types.

### Distributions of breaks/cell

**Fig. 3:**
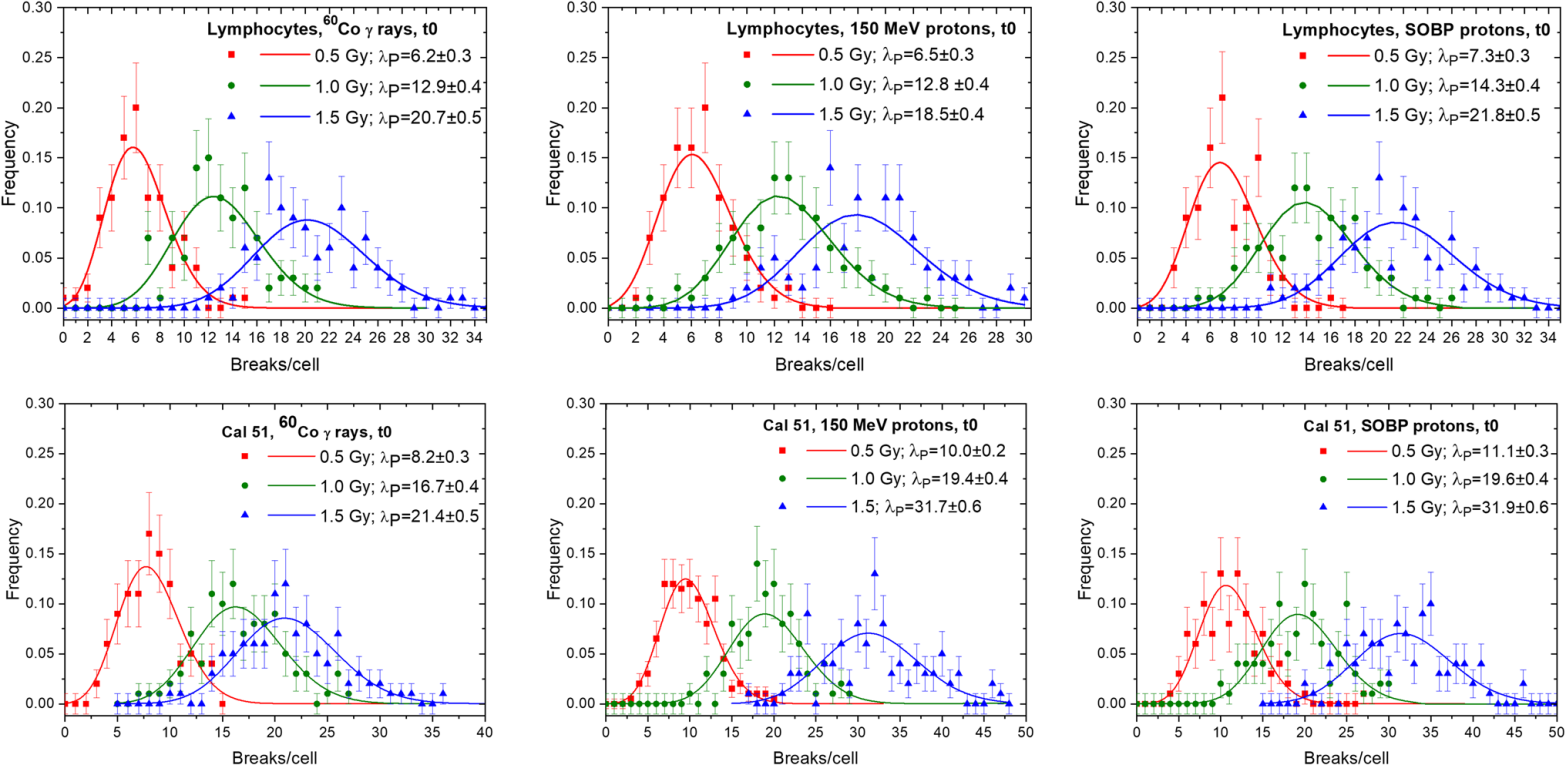
Distributions of PCC breaks/cell measured directly after irradiation (t0) in PBL (upper panel) and Cal 51 (lower panel) exposed to ^60^Co γ-rays, 150 MeV protons and SOBP protons. Data were fitted by Poisson distribution (solid lines) in accordance to the experimentally obtained l_p_

**Fig. 4:**
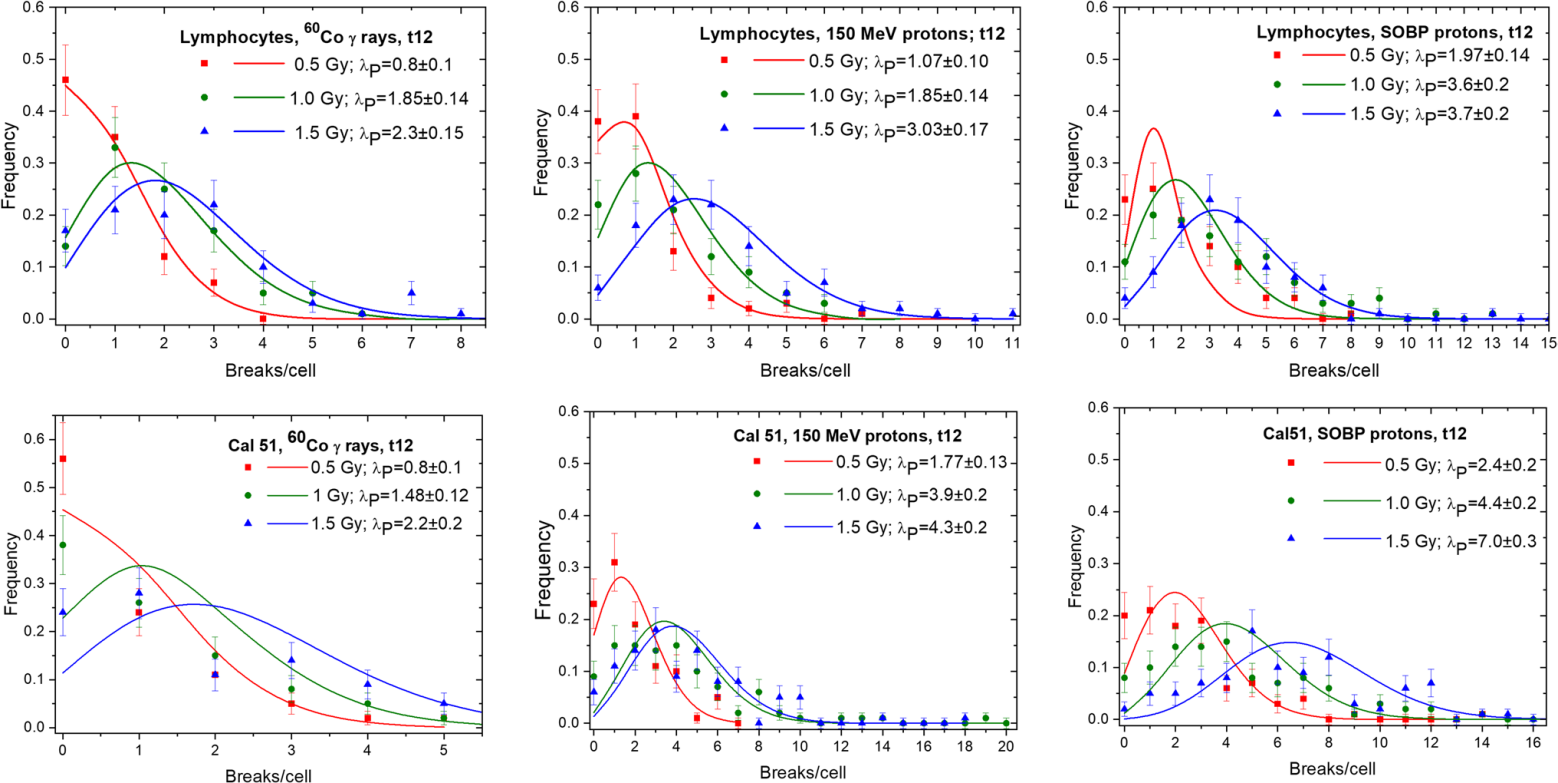
Statistical distributions of PCC breaks/cell measured after repair completion (t12) in PBL (upper panel) and Cal 51 (lower panel) exposed to ^60^Co γ-rays, 150 MeV protons and SOBP protons. Data were fitted by Poisson distribution (solid lines) in accordance to the experimentally obtained l_p_

In Figs. 3 and 4, the statistical distributions of PCC breaks/cell measured directly after irradiation (t0) and after repair completion (t12), respectively, in both cell lines are presented. For each exposure and cell type (PBL and Cal 51), three doses were analyzed: 0.5 Gy, 1 Gy and 1.5 Gy. All distributions are well described using the Poisson statistics. In the case of ^60^Co γ-exposure, the mean number of breaks/cell induced in both cell lines are very similar for each examined dose. The difference is, however, significantly higher for both proton beams: 1.5 Gy of 150 MeV and SOBP protons induced in Cal 51 on average 31.7 ± 0.6 and 31.9 ± 0.6 breaks/cell, respectively, and in PBL 18.5 ± 0.4 and 21.8 ± 0.5 breaks/cell, respectively (Fig. 3). The maximum number of breaks detected in a single cell exposed to 1.5 Gy of both proton beams was also higher for Cal 51 cells, reaching up to 40 and 45 breaks per cell (150 MeV and SOBP protons, respectively). For comparison, for the same dose, the maximal number of breaks per cell detected in PBL was 30 (150 MeV protons) and 33 (SOBP protons).

Twelve hours after irradiation (Fig. 4), the number of PCC breaks/cell dropped down drastically, reflecting the efficient repair in both cell lines. Mean number of breaks/cell (λ_P_) calculated for ^60^Co γ-rays do not differ significantly between PBL and Cal 51. In contrast, for both proton beams the residual mean numbers of breaks/cell were significantly higher for Cal 51 as compared to PBL, the maximal registered values amounted 18 breaks/cell for Cal 51 and 8 breaks/cell in PBL (at 1.5 Gy SOBP protons).

### Ratio of the number of breaks at t0 and t12

**Fig. 5:**
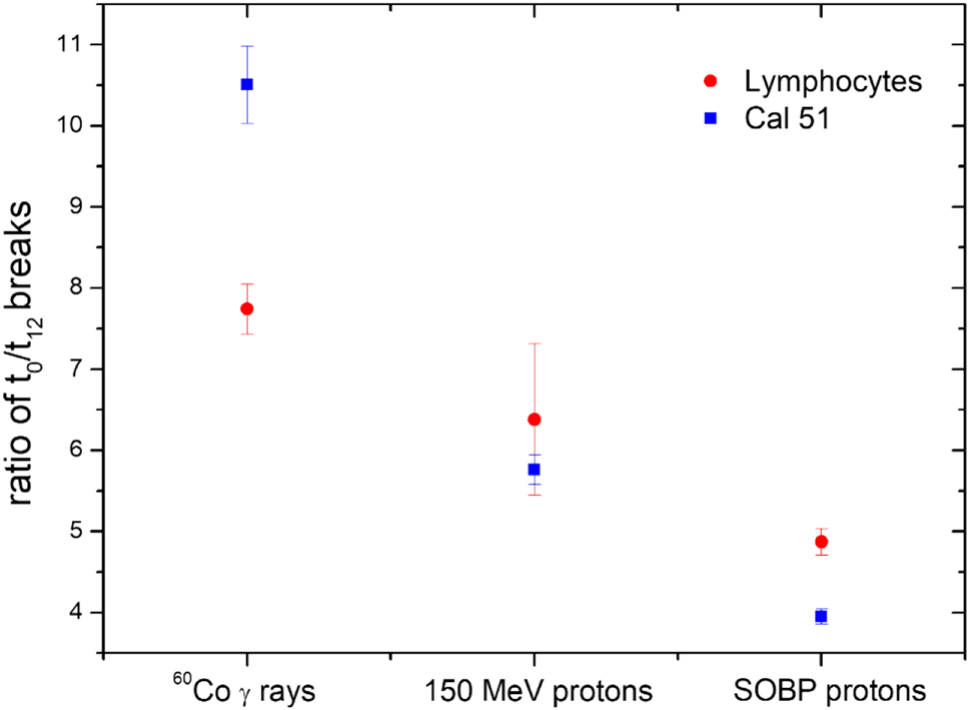
The efficiency of repair of PCC breaks estimated as the ratio t0/t12 of the mean number of breaks/cell, calculated in PBL and Cal 51. Indicated are mean values obtained for four irradiation doses (0.5 Gy, 0.75 Gy, 1.0 Gy and 1.5 Gy). Error bars represent Poisson standard deviations

In order to assess the repair efficiency of normal vs. cancer cells, the ratio of breaks measured at t0 and t12 was calculated (Fig. 5). For each analyzed cell line, radiation type and time point (t0 or t12) the mean number of breaks/cell obtained for four irradiation doses (0.5 Gy, 0.75 Gy, 1.0 Gy and 1.5 Gy) were calculated and used to estimate the t0/t12 ratio. Including all the breaks measured for four doses reduced the statistical uncertainties. After photon exposure, the efficiency of repair is statistically significantly higher for Cal 51 while after proton exposures the repair efficiency is statistically higher in PBL. Efficiency of repair clearly decreases with LET for both cell types, and this effect is more pronounced for tumor cells.

### RBE

**Fig. 6:**
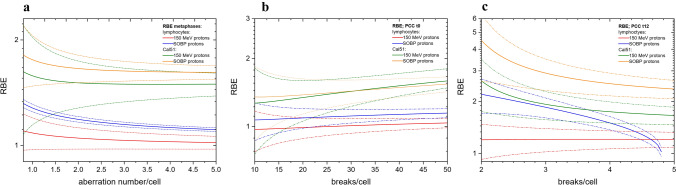
RBEs of SOBP and 150 MeV protons as a function of: **a** chromosome aberration number/cell induced in PBL and Cal 51 cells; **b** PCC breaks/cell induced in PBL and Cal 51 cell immediately after the exposure (t0); **c** PCC breaks/cell induced in PBL and Cal 51 cell 12 h after the exposure (t12). Solid lines represent RBE values; dashed lines of a particular color reflect uncertainty of the corresponding RBEs

In Fig. 6, the RBE of both proton beams obtained in PBL and Cal 51 is presented. The corresponding values were calculated using the parameters of the LQ model and shown as a function of the number of chromosome aberrations per cell for the metaphase assay (Fig. 6a) or as a function of PCC breaks per cell observed just after the exposure (t_0_, Fig. 6b) or 12 h later (t_12_, Fig. 6c). The uncertainties of RBE (1σ confidential level) are presented by means of dashed lines.

Comparison of both proton beam RBE values based on CA yields in metaphases of both cell lines (Fig. 6a) reveals: 1) systematically higher RBE for the SOBP beam compared to fast protons; 2) significantly higher RBE of protons in cancer cells: 1.02 ± 0.04 vs. 1.50 ± 0.11 for 150 MeV protons and 1.11 ± 0.01 vs. 1.61 ± 0.04 for SOBP protons at the level of 5 CA/cell in PBL and Cal 51, respectively.

In Fig. 6b, RBE values as a function of t0 PCC breaks per cell are presented. For Cal 51, the RBE values are slightly higher than for PBL. However, a significant difference is observed only for a higher level of damage, above ~ 30 breaks per cell. In PBL, RBE of fast protons ranges from 1.0 ± 0.2 for 10 breaks per cell to 1.04 ± 0.05 for 50 breaks per cell. RBE of SOBP protons amounts to 1.1 ± 0.2 for 10 breaks per cell and 1.15 ± 0.05 for 50 breaks per cell. In the case of Cal 51, RBE of fast protons increases from 1.3 ± 0.6 for 10 breaks per cell to 1.6 ± 0.4 for 50 breaks per cell; RBE of SOBP beam amounts to 1.4 ± 0.5 and 1.54 ± 0.11, respectively. Differences between both proton beams are statistically not significant due to relatively large experimental uncertainties.

Similar tendency is also observed for measurements performed at t12 (see Fig. 6c) although the RBE uncertainties are considerably larger compared to the t0 case. Especially, the negative *β* parameter for SOBP protons in human lymphocytes does not allow to assess RBE for more than 4.8 breaks per cell. The RBE values determined at the level of 4 breaks per cell are as follows: 1.21 ± 0.14 vs. 1.7 ± 0.2 (fast protons) and 1.50 ± 0.13 vs. 2.5 ± 0.4 (SOBP protons) for PBL and Cal 51, respectively. They are considerable larger than those obtained for the metaphase and t0 analyzes.

## Discussion and conclusions

Recently we reported on chromosome damage induced in PBL by different radiation types, as obtained by conventional metaphase assay (Kowalska et al. 2017, 2019) and chemically induced PCC (Kowalska et al. 2020). As an extension of our studies, the CA yield and PCC induction and repair were investigated in human carcinoma cell line Cal 51 exposed to photon and proton beams which are commonly used for the breast cancer radiotherapy. The main purpose of the present work was to compare the radiation response and efficiency of repair mechanisms of healthy and cancer cells. Cal 51 human carcinoma cell line was chosen for the cytogenetic study owing to perfect diploidy, stable karyotype (Davidson et al. 2020, and own mFISH observations) and low spontaneous CA level which does not exceed 1%. These advantages allow a quantitative comparison of chromosomal radiosensitivity of tumor and healthy human cells which may improve the prediction of radiotherapy outcome.

Both CA and PCC breaks were analyzed by means of the statistical distributions of damage and the corresponding linear-quadratic (LQ) model. Whereas the statistical distributions confirm generally stochastic character of damages described by the Poisson statistics (Kowalska et al. 2019), the LQ parameters can provide direct information about the radiosensitivity and repair efficiency of chosen cell lines. The *α* and *β* parameters, their kinetics in dependence on LET and cell type have also a large impact on the modeling of the cancer radiotherapy treatment outcomes. Whereas the linear term is proportional to the LET value of the particle radiation and corresponds to the number of the DNA double-strand breaks induced, the quadratic term mainly has biological origin and can be related to very efficient DNA repair mechanisms which strongly depend on the local ionization density and thus, on the radiation quality (Kowalska et al. 2017, 2019, 2020; Scholz 2006).

These relations were also confirmed in our present study. For chromosome aberration induction in both cell types, the highest α values are observed for SOBP protons and the smallest for g-irradiation (see Table 2). Furthermore, the α values obtained for Cal 51 cells are higher than for PBL, confirming their higher radiosensitivity. The ratio of these coefficients ($${\raise0.7ex\hbox{${\alpha_{{{\text{Cal}}51}} }$} \!\mathord{\left/ {\vphantom {{\alpha_{{{\text{Cal}}51}} } {\alpha_{{{\text{PBL}}}} }}}\right.\kern-0pt} \!\lower0.7ex\hbox{${\alpha_{{{\text{PBL}}}} }$}}$$) obtained for Cal 51 cells and PBL is the highest in the case of ^60^Co γ-rays and amounts to 11.6 ± 1.8 and decreases with increasing LET, amounting to 7.3 ± 1.9 and 4.5 ± 0.9 for 150 MeV and SOBP protons, respectively. It means that the increase of the radiosensitivity with the LET value is weaker for Cal 51 than for PBL. On the other hand, the quadratic coefficient β does not depend strongly on the LET values studied, which confirms our previous finding (Czerski et al 2019), though the β values except ^60^Co γ-rays are systematically ~ 30% larger for human carcinoma cell line.

In order to determine the repair efficiency, the β/α ratios should be estimated (see Tables 2, 3, 4). In the metaphase assay (Table 2), we have observed higher β/α ratio for lymphocytes compared to Cal 51 cells for all radiation exposures. It might lead to the conclusion that the chromosome damage in PBL can be repaired more effectively. The difference may be, however, partially attributed to irradiation scheme. Lymphocytes were exposed in resting state (G_0_ phase of cell cycle), and Cal 51 as asynchronously growing population. Thus, the direct comparison of CA induced in G_0_-irradiated lymphocytes and in asynchronously growing carcinoma cells is not fully correct due to different sensitivity of G_0_- and G_1_-S-G_2_-irradiated cells. G_0_ cells have more time for repair, and another repair mechanisms dominates at different cell cycle stages: more error prone NHEJ is predominant in G_1_/G_0_ and early S-phase, whereas HR in the S- and G_2_-phase of the cell cycle (Budman and Chu 2005; Grosse et al. 2014). According to the finding of Savage (1975), the closer to mitosis the higher aberration yield and the lower exchange portion in total CA yield. We have also observed differences in aberration spectrum within the two studied cell lines. Irradiation of PBL in G_0_ resulted in > 98% chromosome-type aberrations with 60–80% of exchange-type aberrations (Kowalska et.al. 2019) while irradiation closer to mitosis mainly results in breaks: Cal 51 have about 50% of exchange-type aberrations and half of them are of chromatid type (Table 1). This is a sign that majority of Cal 51 cells were in S (G_1_-S-G_2_) at irradiation time. In addition, there is a large difference in the nucleus sizes and geometry of the studied cells: the spherical compact heterochromatin nuclei of G_0_ PBL are much smaller than the flat ellipsoidal euchromatin nuclei of Cal 51.

In contrast, for PCC analysis, both cell lines were treated under the same conditions as asynchronously growing populations. Equal experimental conditions in the case of PCC allow the direct comparison of chromosome breakage and repair. However, the β/α ratios obtained for the PCC study have very large statistical uncertainties, and, therefore, any direct comparison between the two cell lines is not possible (Tables 3–4). Fortunately, in the case of PCC, another method can be applied for estimation of the repair efficiency. The approach proposed is based on the determination of the total number break ratio observed at t0 and t12 radiation exposure (Fig. 5): the lower the ratio, the less efficient repair. According to Fig. 5, it is clear that this ratio is decreasing with LET and the decrease is much stronger for Cal 51 cells. Furthermore, the most pronounced decrease is observed for Cal 51 cells after SOBP proton exposure. This is an important finding of the present work, strongly supporting the use of the proton therapy for breast cancer patients.

This finding can be also supported by the analysis of RBE functions (Fig. 6a–c). The RBE values determined for Cal 51 cells are systematically higher than for PBL, with the maximum seen in the t12 study, where the RBE = 2.5 ± 0.4 for SOBP protons at the level of 4 breaks per cell was observed. In particular, the residual damage observed in chromatin after repair completion accounts for the biological effectiveness of radiation. In addition, the biological efficiency of the SOBP beam, which is also used in cancer treatment, was significantly higher than that of the fast protons, confirming the LET dependence of the RBE values (Nasonova et al. 2001, Deperas-Standylo et al. 2012).

In summary, we have conducted—for the first time to our knowledge—an investigation of breast cancer cell vs. PBL chromosomal radiosensitivity following photon and proton exposures using metaphase and PCC analysis. Both cytogenetic assays confirmed the higher efficiency of proton beams in tumor cells compared to PBL: Cal 51 cells have more efficient repair after photon treatment than PBL cells, but were shown to be more sensitive to protons. The lower DNA repair capacity in Cal 51 cell line after proton irradiation may be caused by defects in the DNA repair pathways, particularly homologous recombination. Grosse and co-workers (Grosse et al. 2014) have shown that the lack of HR proteins leads to higher sensitivity to proton than to photon irradiation. In addition, proton beams have higher potential to eliminate cancer stem-like cells (Schniewind et al. 2022) and cause stronger suppression of molecular and cellular processes (i.e., cell adhesion, migration ability and apoptotic rate) that are fundamental to tumor expansion (Fu et al. 2012; Narang et al. 2015; Zhang et al. 2013). These findings, together with the fact that Cal 51 cells tolerate photon exposure but are more sensitive to protons, support the use of protons in radiotherapy for breast cancer patients.

## Data Availability

The datasets generated during and/or analysed during the current study are available from the corresponding author on reasonable request.
